# Enflicoxib for canine osteoarthritis: A randomized, blind, multicentre, non-inferiority clinical trial compared to mavacoxib

**DOI:** 10.1371/journal.pone.0274800

**Published:** 2022-09-20

**Authors:** Marta Salichs, Llorenç Badiella, Patxi Sarasola, Josep Homedes

**Affiliations:** 1 Ecuphar Veterinaria SLU (Animalcare Group) Sant Cugat del Vallès, Barcelona, Spain; 2 Servei d’Estadística Aplicada, Universitat Autònoma de Barcelona, Bellaterra, Barcelona, Spain; 3 Ondax Scientific SL, Hondarribia, Spain; Keio University School of Medicine, JAPAN

## Abstract

**Background:**

This prospective, multisite, blinded, randomized, non-inferiority clinical study aimed to confirm the efficacy and safety of enflicoxib in the treatment of pain and inflammation associated with canine osteoarthritis. A total of 180 dogs were randomized to receive enflicoxib (n = 78), mavacoxib (n = 80) or placebo (n = 22). Dogs underwent veterinary assessments from day 0 to day 42 using a clinical sum score (CSS). Efficacy was also assessed by the owners using the Canine Brief Pain Inventory (CBPI). The primary efficacy endpoint was the overall CSS from day 0 to day 42.

**Results:**

The overall CSS expressed as area under the curve demonstrated non-inferiority of enflicoxib compared to mavacoxib, and both showed superiority over placebo. At the end of the study, average CSS, and the percentage of CSS responders for enflicoxib (3.64 and 74%) and mavacoxib (4.49 and 68%), was superior to placebo (7.15 and 29%). A faster onset of action was observed for enflicoxib as superiority over placebo was evidenced from the first efficacy assessment (day 7) onwards for both parameters, whereas mavacoxib was only significantly different from day 14 onwards. According to the owner assessment, the percentage of CBPI responders was 90%, 79%, and 43% for dogs treated with enflicoxib, mavacoxib and placebo, respectively, and superiority over placebo was demonstrated for both active treatments. In all secondary parameters, non-inferiority of enflicoxib versus mavacoxib was confirmed. The dog’s quality of life improved in all groups, but only enflicoxib showed superiority versus placebo. When assessing severely affected dogs only, results were similar, thus confirming the efficacy of enflicoxib in all stages of canine OA. There were no differences between groups in the frequency of adverse events, which were most frequently mild affecting the gastrointestinal tract and recovered without treatment.

**Conclusions:**

Enflicoxib is efficacious and safe for the treatment of pain and inflammation in any stage of canine osteoarthritis with a faster onset of action compared to mavacoxib.

## Introduction

Osteoarthritis (OA) is a highly prevalent progressive degenerative disease of synovial joints [1–5] with 20 per cent or more of the canine population over the age of one year old affected by the disease [6–9]. This musculoskeletal disease results in lameness, loss of joint function and mobility, chronic pain, and reduced quality of life [10]. The management of OA in dogs is a lifetime commitment, involving a multimodal approach based on relieving the symptoms of the disease by treating pain and inflammation, improving mobility and hence quality of life, whilst protecting joints from OA [11–15]. Non-steroidal anti-inflammatory drugs (NSAIDs) have been the medical cornerstone for the management of pain and inflammation in canine OA for many years [16, 17] and preferential and selective cyclooxygenase-2 (COX-2) inhibitors have been developed to potentially reduce the risk of unwanted side effects caused by the inhibition of COX-1 [18, 19]. Further to classic NSAIDs therapy, newly registered products such as grapiprant and bedinvetmab have also shown efficacy in the control of pain associated to OA [20, 21].

The pain associated with OA is chronic [22] and long-term continuous NSAID treatment has been shown to be more efficacious than short-term treatment periods, with no evidence of any increase in NSAID related side effects [23]. However, side effects may occur and should be monitored [24–27].

All available NSAIDs have demonstrated similar efficacy in reducing pain related to OA. However, most NSAIDs require daily oral administration to ensure their efficacy and therefore a substantial number of dog owners and caregivers are charged with the responsibility of managing their treatment [28]. In this sense, compliance with long-term daily administration of medicines in routine veterinary clinical practice is known to be relatively poor and a major barrier to adequate treatment [29], with daily doses being missed even during a relatively short treatment course [30, 31]. Therefore, simplifying dosing with long-acting products with less frequent dosing, likely to achieve an increased overall compliance [32], may be more reliable to treat chronic pain for some dogs and their owners [21].

Enflicoxib, is a new selective COX-2 inhibitor with long-lasting activity in dogs that has recently been registered in Europe for the treatment of pain and inflammation associated with OA in dogs [33]. Enflicoxib efficacy is achieved through its active metabolite which, after repeated weekly administrations, achieves blood levels that remain stable within its therapeutic window [34–36].

In a previous clinical study, enflicoxib has shown to be superior to a placebo, with a good safety profile, at a dose of 4 mg/kg, once a week, with an initial loading dose of 8 mg/kg [37]. The present study follows very similar procedures to the former one with the objective to confirm the clinical relevance of the selected enflicoxib dose, by demonstrating non-inferiority to an approved and effective NSAID for the treatment of canine OA (mavacoxib, Trocoxil®, Zoetis) that is used as reference product.

## Materials and methods

This prospective, multisite, blinded, randomized, controlled, parallel-group non-inferiority field study was conducted in compliance with the Veterinary International Conference on Harmonization guideline for Good Clinical Practice [38] at 20 veterinary practices located throughout Spain and France. Approval was obtained from the Spanish Agency for Medicines and Medical Devices (AEMPS), with protocol number 357/ECV, and by the French Agency for Veterinary Medicinal Products (ANSES), with number EC-00769-0, and satisfied national regulatory and animal welfare standards and requirements. Written informed consent was obtained from all dog owners prior to enrolment of their dogs in the study. Dogs remained under the care of their owners at home during and after the study.

### Inclusion and exclusion criteria

Client-owned dogs of both sexes and any breed presented as veterinary patients showing clinical signs of OA such as pain and lameness for at least 3 weeks were evaluated and scored for possible inclusion in the study. Radiographic evidence of OA in the joint where signs of pain were present (presence of articular lesions compatible with OA, such as subchondral bone sclerosis, bone remodelling, osteophytes, irregular or diminished joint space) was required.

Prior to inclusion in the study, dogs should not have received any treatment with short acting NSAIDs, corticosteroids or opioids for at least 14 days, pentosan polysulphate sodium, PSGAG (polysulphated glycosaminoglycan), long-acting systemic corticosteroids or mavacoxib for at least 30 days, or intra-articular injections of corticosteroids for 90 days. Additionally, dogs should not have received chondroitin sulphate or glucosamine or a specific OA prescription diet containing chondroprotective agents, except if these products had been administered at a constant dosage for at least one month before the start of the present study and administration would not be altered during the study. Dogs known to have severe or uncontrolled concomitant disorders (e.g. kidney, liver, heart, gastrointestinal tract, or haemorrhagic disorders including hypovolemic, dehydrated, hypotensive or unexplained bleeding episodes) that are contraindications for the use of NSAIDs, or that could interfere with the evaluation of treatment effect, were excluded from participation. Dogs in which surgery had been performed on any joint in the previous 60 days or with axial skeleton disease, or in which the presenting lameness was associated with active infectious arthritis, neoplasia, a primary neurological disorder or known immunological disorder, were also excluded. Dogs were not eligible for enrolment if gross instability of the hip or the stifle joint was present. Females that were pregnant or lactating, or animals intended for breeding were not included.

Concomitant treatment with analgesic drugs, NSAIDs or systemic corticosteroids was not permitted during the study. Administration of other concomitant medications was permitted but had to be recorded. Dogs with mild and controlled conditions could participate, and their medication could be continued, if it was not expected to alter the study results. Other limitations included in the summary of product characteristics of mavacoxib were also implemented.

The severity of clinical signs of OA was evaluated by both the veterinarian and the dog owner on the day of inclusion, before first treatment administration.

The veterinarians assessed pain and lameness using NRS as described by several authors [39–43]. This NRS included the assessment of four parameters in the following order: posture while the dog was standing, lameness at walk, lameness at trot and pain at palpation/manipulation of the affected joint as described in Salichs et al. [37]. A factor of two was applied to place more weight on lameness at walk and at trot as part of the clinical picture of OA [39, 40, 43]. The clinical sum score (CSS) was the sum of scores for these four parameters and ranged from 0 to 18.

The dog owner assessments of pain and quality of life was performed using the canine brief pain inventory (CBPI) [44, 45]. The CBPI is a 2-part instrument: the pain severity score (PSS) is the arithmetic mean of 4 questions scored on an 11-point (0 = no pain to 10 = severe pain) numerical scale, and the pain interference score (PIS) is the mean of 6 questions scored similarly (0 = no interference to 10 = severe interference) to evaluate the pain interference with the dog’s general activity, enjoyment of life and locomotive function. In addition, the owner was asked to also rate his or her overall impression of the dog’s quality of life, which was graded as Poor, Fair, Good, Very Good or Excellent.

Dogs selected for inclusion in the study had to have clinical signs of OA as evidenced by a CSS≥6 and PSS and PIS scores ≥ 2 on Day 0 prior to treatment. All dogs included in the study had to be in good general health based on a complete general physical examination, and routine blood (haematology and biochemistry) examination results within normal limits. Dog owners were instructed not to change, as far as possible, the daily exercise routine or home management of their dogs during the study in order not to have an impact on the evaluation of efficacy of the test products.

Any dog could be withdrawn from the study in case of occurrence of an adverse event that required stopping the treatment or which could interfere with the evaluation of the study treatment; an unsatisfactory therapeutic effect; forbidden concomitant treatment; a major protocol deviation, or withdrawal of the owner’s consent. For cases with unsatisfactory therapeutic response, additional veterinary care including rescue analgesia was permitted after withdrawal of the dog from the study.

### Treatments

Dogs that met the inclusion criteria were enrolled by the veterinarians and randomly allocated to one of three oral treatment groups following a randomisation list with an allocation ratio of 4:4:1 for active treatments and placebo. The smaller negative control group was included to establish superiority of both treatments to placebo and hence validate the study design. Day 0 was defined as the day of inclusion and the first day of treatment. Each dog received treatment with enflicoxib (Daxocox®, Ecuphar/Animalcare group) at once weekly maintenance dose of 4 mg/kg on days 7, 14, 21, 28 and 35 with an initial loading dose of 8 mg/kg on Day 0, or mavacoxib (Trocoxil®, Zoetis) at 2 mg/kg on Day 0 and Day 14, according to its summary of product characteristics, with the administration of a placebo tablet on days 7, 21, 28 and 35 to ensure blinding. Animals in the negative control group received a placebo tablet on days 0, 7, 14, 21, 28 and 35.

Enflicoxib and mavacoxib had a different appearance. Therefore, to preserve blinding of the investigator and the owner, a dispenser was identified at each site for the allocation, administration and dispensing of study treatments. The random allocation was implemented using sequentially numbered containers. Treatment on days 0, 7, 14 and 28 was administered at the veterinary practice by the dispenser, while treatment on days 21 and 35 was administered by the owner at home. Dose calculations for study treatments were performed using the body weight determined on Day 0. As food increases its absorption, and following their label indications, enflicoxib, mavacoxib or placebo tablets were administered with food or immediately before feeding [34, 46]. At the end of the study, the owner assessed the general level of acceptance by the animal of the treatments given at home as poor, satisfactory, good, or excellent.

### Assessments

General physical examinations and clinical assessments of pain and lameness were performed by the veterinarian on Day 0, prior to treatment and thereafter at each study visit on days 7, 14, 28 and 42 (±2 days) using the CSS. The most severely affected joint was selected on Day 0, prior to the start of treatment administration and evaluated throughout the study regardless of whether another joint was also affected. In addition, during each clinical assessment on days 7, 14, 28 and 42 and through a telephone call on days 21 and 35, the veterinarian interviewed the owner to record their assessments using the CBPI. The owner was not aware of the required threshold level for PSS and PIS scores for inclusion in the study and did not have access to the scores of previous assessments when completing each CBPI.

### Efficacy outcome measures

Scores for each of the categories within the veterinary assessment (CSS) and the owner assessment (CBPI) were calculated for each dog for each assessment day. For each assessment after Day 0 the percentage of responders to treatment according to each parameter (CSS and CBPI) was determined using a predefined criterion of treatment response. For the veterinary assessment, a dog was classified as a responder if the CSS score was <6, and for the owner assessment, a dog was classified as a responder if it had a decrease ≥1 in PSS, and ≥2 in PIS compared to basal scores.

The primary efficacy endpoint was based on the veterinary assessment for the overall CSS (the CSS area under the curve (AUC) calculated from Day 0 to Day 42):

$$AUC=\sum_{i=0}^{I}\frac{\left(CSS_{i}+CSS_{i-1}\right)}{2}(t_{i}-t_{i-1})$$,

where *I* represents the number of time points, *t_i_* is the ith time point and *CSS_i_* corresponds to CSS value at the ith time point.

Secondary efficacy endpoints also related to the veterinary assessment were, the total CSS at each assessment and the percentage of CSS responders at the end of the study (Day 42) and on days 7, 14 and 28. The percentage change in total CSS from Day 0 to Day 42 was also calculated.

Secondary efficacy endpoints related to the owner assessment were, the PSS and PIS at each time point, as well as the percentage of CBPI responders at the end of the study (D42) and on days 7, 14, 21 and 28.

Any dog not classified as a responder using the above-mentioned criteria or withdrawn from the study because of lack of efficacy prior to Day 42 was classified as a treatment failure. The treatment failure classification and the clinical scores at the time of withdrawal from the study were carried forward to all subsequent time points subjected to the Last Observation Carried Forward (LOCF).

Moreover, the overall Owner impression of the dog´s quality of life was compared between treatments at each time point.

In all parameters, superiority over placebo and non-inferiority over mavacoxib, using a 15 per cent non-inferiority margin, was statistically assessed.

To obtain specific information on the efficacy of the treatments in severe osteoarthritis cases, a subgroup of dogs having a basal CSS≥8 was selected and further analysed following the same statistical approach.

### Safety outcome measures

Safety was evaluated by recording AEs that occurred throughout the study. Owners were informed about the most common AEs related to NSAID administration such as vomiting, melena, diarrhoea, anorexia, and were instructed to daily observe the animals and to immediately report any suspect AE to the veterinarian. An AE being defined as any observation in animals that is unfavourable and unintended and occurs after the use of enflicoxib or mavacoxib, whether or not considered to be product related [47]. Each AE was described by clinical signs using the Veterinary Dictionary for Drug Regulatory Activities (VeDDRA) terms [48]. The severity of the clinical signs (mild, moderate, severe) and the outcome of the AE, and whether it was serious or not was indicated. A Serious Adverse Event (SAE) was considered an AE that results in death, is life-threatening, results in significant disability or incapacity, is a congenital anomaly/birth defect, or results in permanent or prolonged signs. By default, any AE not falling into the definition of SAE is considered “non-Serious”. At the end of the study, all AE were assessed using the ABON system of causality assessment [49], where A = probable, B = possible, O = unclassifiable/unassessable, O1 = inconclusive and N = unlikely to be treatment related. This assessment considered that NSAIDs have the potential to cause or exacerbate gastrointestinal, renal, and hepatic disorders.

For the calculation of the incidence, when several AEs were observed in a single animal at an overlapped time frame, according to current guidelines [50] they were considered as different clinical signs of the same AE.

### Data management

The study data was recorded contemporaneously by the investigators and dispensers in electronic Case Record Forms. The validated study specific Electronic Data Capture database was designed and validated by Ondax Scientific using the Ennov® system as described in the Data Management Plan. Data was subjected to 100% quality control checks. Following data query resolution and database quality audit, the database was locked, and data exported in.sas and.dat data files for statistical analysis.

### Sample size

The primary variable is defined as the average AUC from 0 to 42d of CSS. Based on data from previous internal studies, the expected average for the active groups was set to 240 units, with an estimated standard deviation of approximately 85. Considering that the average for the placebo was approximately 320 points, the margin on non-inferiority was set to 36 points, which represents a margin level of 15% with respect to the reference group. In order to demonstrate the non-inferiority of the experimental treatment with respect to the reference treatment, a sample size of 70 animals per group was required in order to achieve a power of 80% when applying a t-test for non-inferiority with a one-sided significance level of 5%. The final sample size was increased to 75 animals per group to prevent for potential withdrawals.

### Statistical analysis

The statistical analysis was performed in two different populations. The Intention To Treat (ITT) population included all animals that were randomized and received at least one dose of study treatments. The Per Protocol (PP) population included dogs that were fully compliant with the protocol except for cases with minor deviations that would not affect the results. As the severity of the disease at the time of enrolment can negatively affect the efficacy outcomes [37], further statistical analyses were carried out on a subset of the PP population including only dogs with initial CSS≥8 to assess the effect of treatment in dogs with more severe clinical signs of OA.

Demographic and baseline data evaluation was carried out on the ITT population. Baseline analyses were considered from a qualitative point of view to evaluate if groups were properly balanced.

The evaluation of the primary and secondary efficacy endpoints was conducted on the PP population, while the safety of the products was performed on the ITT population. Quantitative variables were analysed by means of the appropriate test (ANOVA, Kruskal-Wallis). The compliance of application criteria was assessed by means of Kolmogorov-Smirnov normality test and Levene’s test for homogeneity of variances. For categorical variables, differences between groups were evaluated by means of the appropriate test (Chi-Square test, Fischer’s exact test or LR Chi-Square test). The compliance of application criteria was assessed by means of the Cochran’s rule. These procedures were adapted to evaluate non-inferiority when comparing the experimental treatment to the reference group, obtaining 90% CI for the differences between groups and the correspondent non-inferiority one-sided p-value.

The statistical analysis was performed using SAS System® v9.4 (SAS Institute Inc., Cary, NC, USA). For all statistical tests a nominal significance level of 5% (P <0.05) was applied. No multiplicity correction was applied for secondary endpoints.

## Results

One hundred and eighty dogs were enrolled and included in the Intent-to-Treat (ITT) population. Mean bodyweight of animals on Day 0 was 27.29 kg (±11.74) and it ranged from 5 to 63 kg and the mean age was 9.30 years ranging from 11 months to 17 years. Males and females, entire or neutered were enrolled in each treatment group and dogs were predominantly purebred. Affected joints were predominantly the hip in 43% of cases, elbow in 24% and stifle in 23%. As described in Table 1, all treatment groups were balanced for the basal characteristics of the dogs included in the ITT population, as well as for previous medical conditions and medications, physical examination, limb and joint selected for assessment and the signs related to OA assessed by the veterinarian and the owner (p>0.05). Some dogs had mild and well controlled health conditions unrelated to OA that were considered compatible with the inclusion/exclusion criteria.

**Table 1:** Demographic data and CSS, PSS and PIS basal scores for the ITT population.

	Enflicoxib	Mavacoxib	Placebo
	n = 78	n = 80	n = 21
Sex, n (%)			
male	40 (51.3%)	45 (46.3%)	13 (59.1%)
female	38 (48.8%)	35 (43.8%)	9 (40.9)
Age, years			
mean (SD)	8.96 (3.19)	9.42 (3.51)	10.09 (2.69)
range	1.5–17	0.9–17	5–14
Bodyweight, kg			
mean (SD)	26.85 (11.94)	28.30 (11.49)	25.23 (12.10)
range	6–57	7–63	5–45
Breed, n (%)			
mongrel	20 (25.6%)	23 (28.7%)	2 (9.1)
purebred	58 (74.4%)	57 (71.3%)	20 (90.9%)
CSS			
mean (SD)	9.77 (2.69)	9.96 (2.51)	9.59 (2.40)
range	6–18	6–16	7–15
PSS			
mean (SD)	5.07 (1.62)	5.42 (1.28)	5.27 (1.51)
range	2–9	2–9	1.25–8
PIS			
mean (SD)	5.94 (1.68)	6.08 (1.67)	5.48 (1.54)
range	0.33–10	1.67–9.33	2.50–8.50

CSS: Clinical Sum Score; PSS: Pain Severity Score; PIS: Pain Interference Score; ITT: Intention To Treat; SD: Standard Deviation

**Fig 1:**
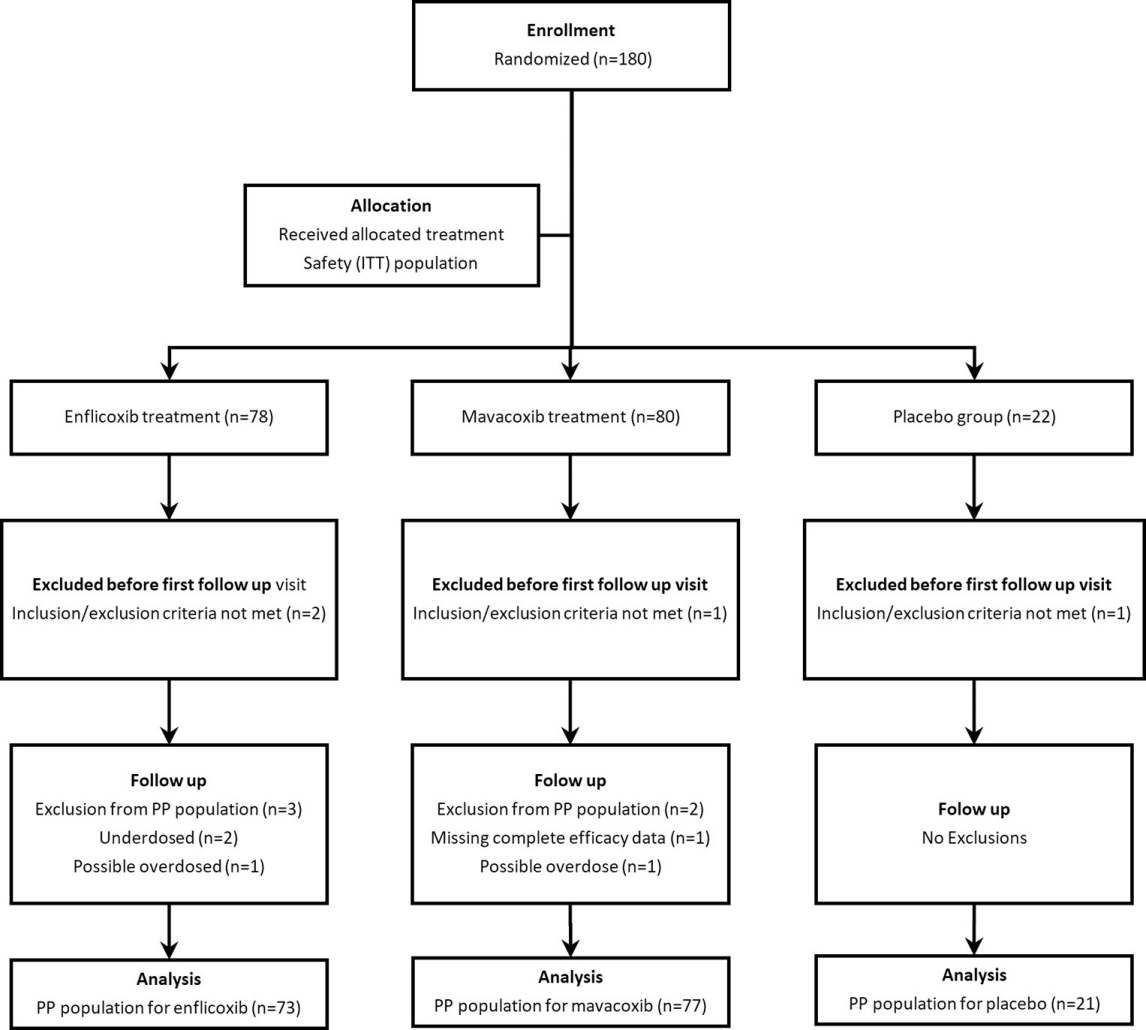
Flowchart showing number of patients recruited, allocated to each treatment, and analysed. (ITT: Intention To Treat; PP: Per Protocol).

Out of this population, nine animals were withdrawn prior to completion of the study, which resulted in a similar Per Protocol (PP) population in each treated group (enflicoxib = 73, and mavacoxib = 77) and 21 in the placebo group (Fig 1).

No dogs were withdrawn due to an apparent lack of efficacy. The population of dogs more severely affected and therefore, complying with having a baseline CSS≥8 on day 0, corresponded to n = 56, 61 and 15 for the enflicoxib, mavacoxib and negative control groups, respectively.

Fifty-six dogs received medications that were administered concurrently with either enflicoxib (24), mavacoxib (25), or placebo (7) during the study. The types of medications included vaccinations, anthelmintic treatments, antimicrobials, topical skin, aural and otitis, as well as flea and tick treatments and products to treat cardiac or gastrointestinal (nausea, emesis, diarrhoea) disorders observed in the adverse events reported during the study.

### Efficacy evaluation

The analysis of the overall CSS values expressed as AUC (217.8±110.9 for enflicoxib, 245.3±111.6 for mavacoxib and 331.8±122.5 for placebo) demonstrated non-inferiority of enflicoxib compared to mavacoxib (Table 2), and both active treatments showed superiority over placebo (p<0.05).

**Table 2:** Non inferiority analysis for the veterinary assessment: CSS; CSS_AUC_; responders’ rate (CSS) and for the owner assessment: PSS, PIS, responders rate (CBPI).

	Enflicoxib	Mavacoxib	Pr<t^‡^
CSS_AUC_ (mean±sd)			
AUC_d0-d42_	217.8±110.9	245.3±111.6	0.0003
CSS (mean±sd)			
CSS_d7_	6.22±3.22	7.04±2.72	< .0001
CSS_d14_	5.05±2.75	5.74±2.98	0.0006
CSS_d28_	4.40±3.05	4.97±3.26	0.0057
CSS_d42_	3.64±3.01	4.49±2.95	0.0011
CSS_responder_ (%)			
CSS<6_d7_	41.10	25.97	0.0005
CSS<6_d14_	54.79	51.95	0.0566
CSS<6_d28_	64.38	62.34	0.0626
CSS<6_d42_	73.97	67.53	0.0139
PSS (mean±sd)			
PSS_d7_	3.91±1.88	4.35±1.57	< .0001
PSS_d14_	3.37±1.83	3.69±1.67	0.0013
PSS_d21_	2.95±1.68	3.31±1.70	0.0011
PSS_d28_	2.82±1.89	3.31±1.91	0.0010
PSS_d35_	2.65±1.90	3.09±1.82	0.0017
PSS_d42_	2.38±1.79	2.92±1.77	0.0005
PIS (mean±sd)			
PIS_d7_	4.66±1.96	4.97±2.00	0.0008
PIS_d14_	4.04±1.97	4.15±2.16	0.0162
PIS_d21_	3.44±1.78	3.81±2.03	0.0015
PIS_d28_	3.26±2.05	3.65±2.21	0.0040
PIS_d35_	3.02±1.94	3.55±2.16	0.0010
PSS_d42_	2.79±1.84	3.36±2.08	0.0005
CBPI_responder_ (%)			
PSS_0-7_>0 and PIS_0-7_>1	50.68	37.66	0.0022
PSS_0-14_>0 and PIS_0-14_>1	73.97	58.44	0.0005
PSS_0-21_>0 and PIS_0-21_>1	84.93	66.23	< .0001
PSS_0-28_>0 and PIS_0-28_>1	82.19	67.53	0.0003
PSS_0-35_>0 and PIS_0-35_>1	87.67	75.32	0.0004
PSS_0-42_>0 and PIS_0-42_>1	90.41	79.22	0.0003

^‡^Non-Inferiority limit was set at -0.15 (15%) of the reference product

CSS: Clinical Sum Score; PSS: Pain Severity Score; PIS: Pain Interference Score; CBPI: Canine Brief Pain Inventory; ITT: Intention To Treat; AUC: Area Under the Curve

**Fig 2:**
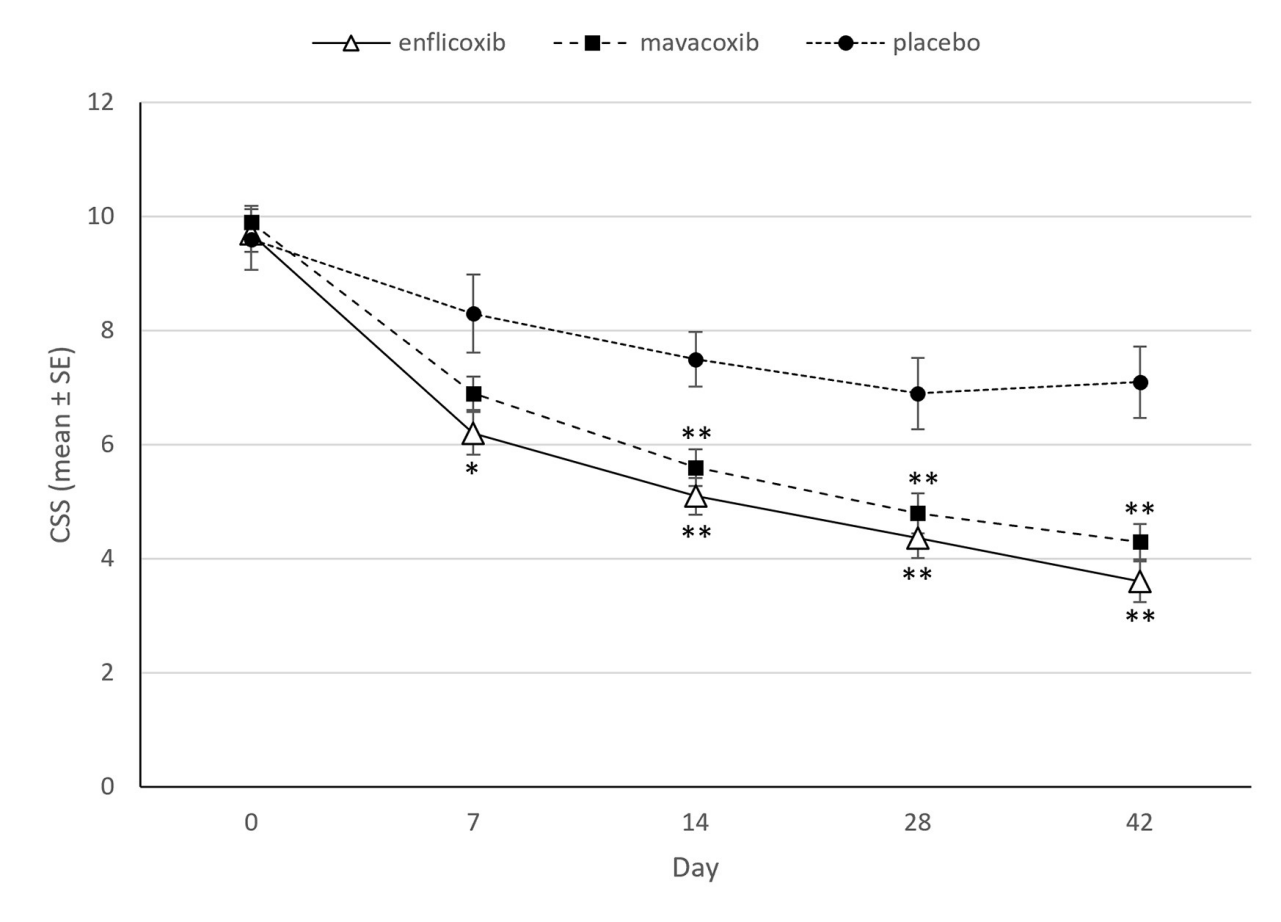
Average CSS (mean±Standard Error) for each time point and treatment. Asterisks indicate superiority vs placebo (*p<0.05 **p<0.01).

The evolution of CSS per treatment group and over time is depicted in Fig 2. The average CSS decreased progressively up to 63 or 57% lower scores on day 42 in the enflicoxib and mavacoxib groups (respectively) compared to baseline. An average 26% decrease was also observed in the placebo group, which was more noticeable in the first two weeks. In both treated groups the total CSS was significantly lower than in the placebo group throughout the study (p<0.05), starting on the first week of treatment for enflicoxib and one week later for mavacoxib. Non-inferiority between enflicoxib and mavacoxib was also demonstrated at all time points (Table 2).

**Fig 3:**
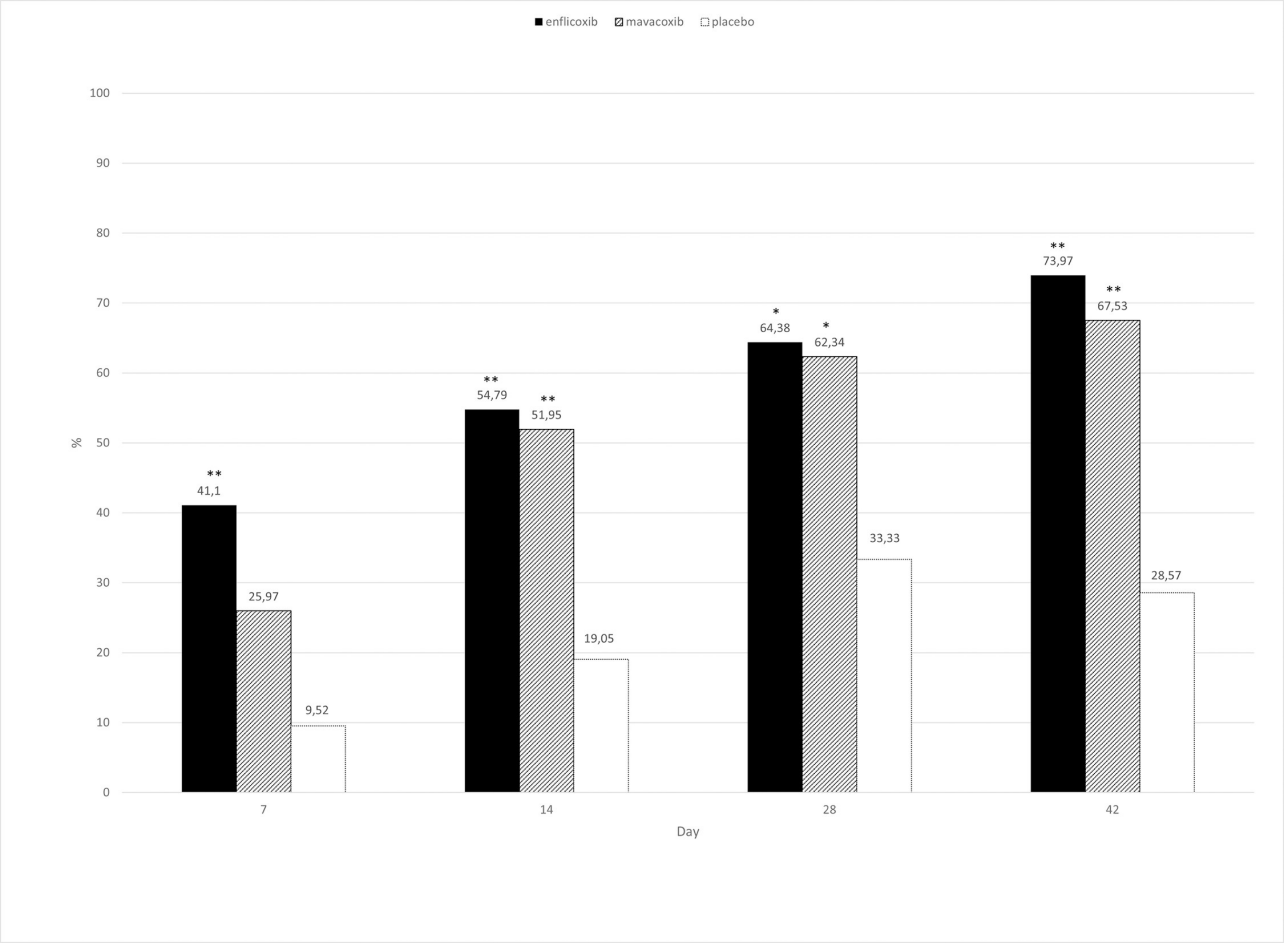
Percentage of CSS responders (CSS<6) in each treatment group and time point during the study. Asterisks indicate superiority vs placebo (*p<0.05 **p<0.01).

According to the CSS responder criteria, at the end of the study, 54/73 dogs treated with enflicoxib responded to treatment, compared with 52/77 of mavacoxib-treated animals. In the placebo group 6/21 were also classified as responders. Fig 3 shows the treatment response rate throughout the study. A rapid response to treatment was observed in enflicoxib treated dogs as this group was statistically superior to placebo from the first assessment at Day 7, whereas mavacoxib treated dogs improved more gradually and this group was only superior to placebo from Day 14 onwards (p<0.05). Non-inferiority of enflicoxib versus mavacoxib was demonstrated at all assessment times (Table 2).

**Fig 4:**
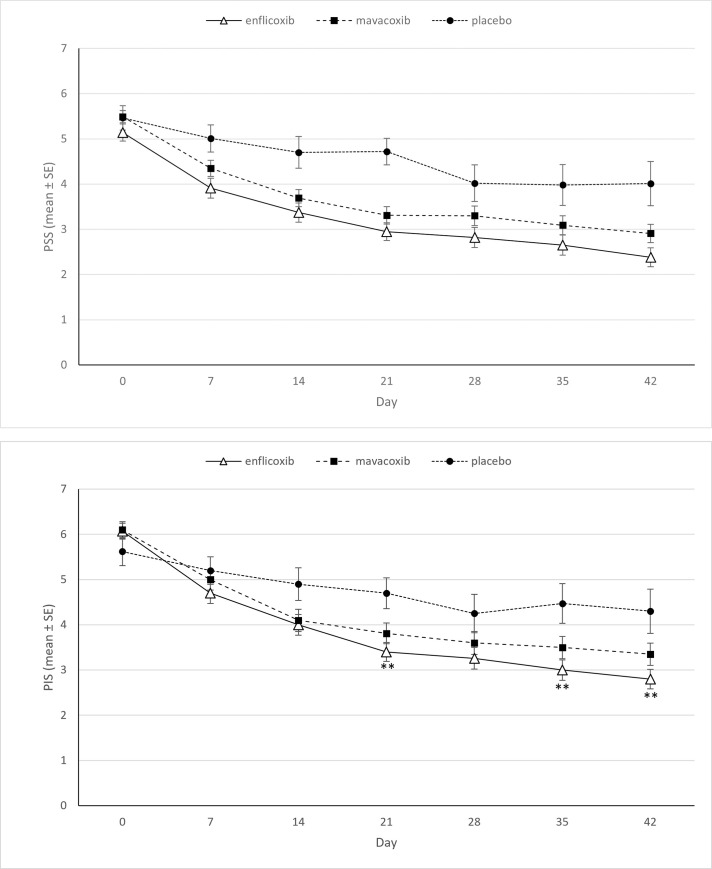
Average PSS and PIS scores (mean±Standard Error) for each time point and treatment. Asterisks indicate superiority vs placebo (*p<0.05 **p<0.01). a) PSS. b) PIS.

The results of the owner assessments using the CBPI reflected those of the veterinarians for the pain severity score (PSS) in the enflicoxib treated group, where superiority versus placebo was observed at all time points (p<0.05). However, after treatment with mavacoxib superiority versus placebo was only observed on days 14, 21 and 42. For the PIS, superiority vs placebo was observed on days 21, 35 and 42 in the enflicoxib treated group, while differences versus placebo were not detected at any time point after treatment with mavacoxib. Non-inferiority of enflicoxib compared to mavacoxib was demonstrated at all time points for PSS and PIS (Table 2). The evolution of PSS and PIS per treatment group and over time is depicted in Fig 4.

**Fig 5:**
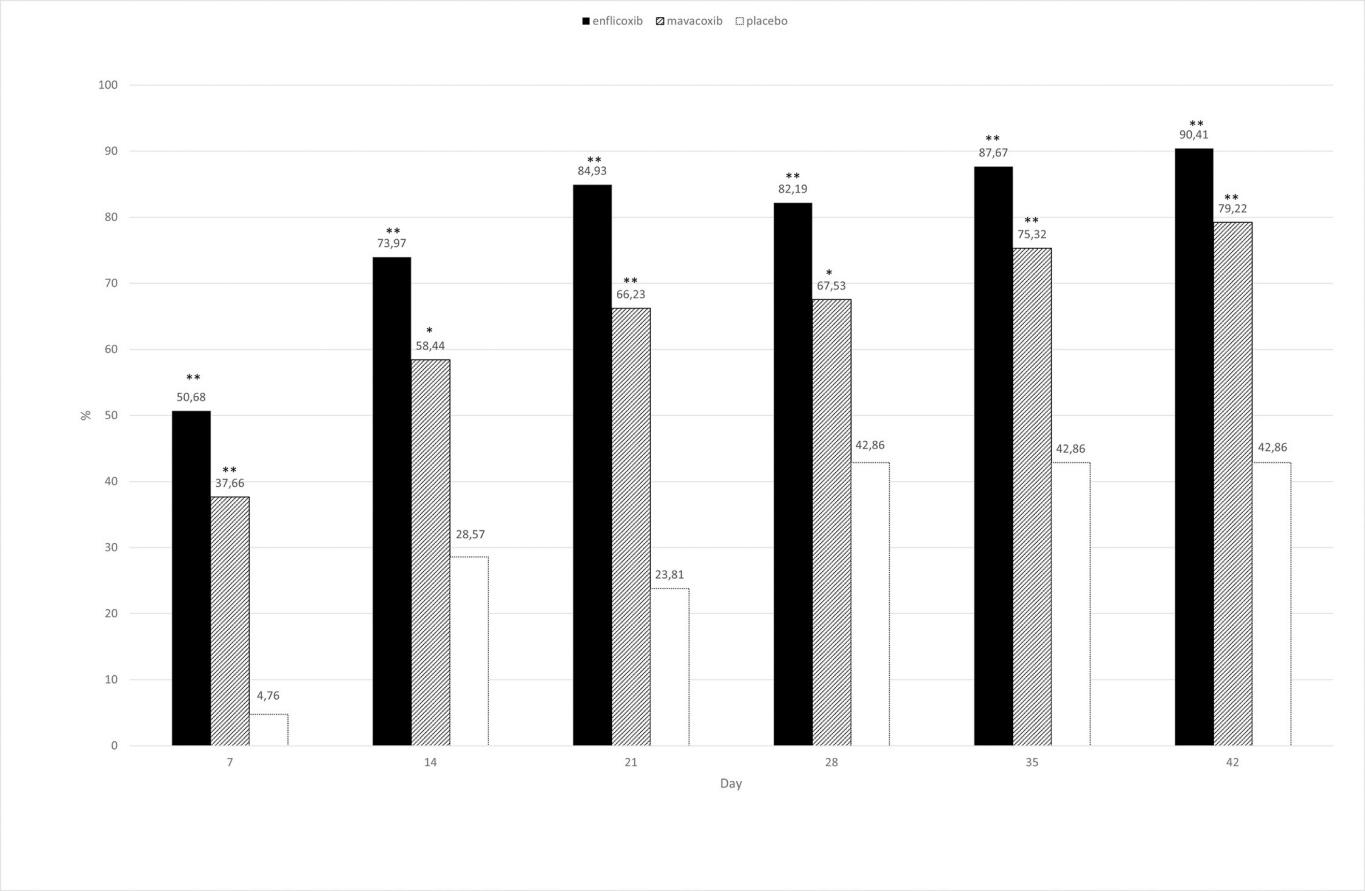
Percentage of CBPI responders (reduction in PSS≥1 and PIS≥2) in each treatment group and time point during the study. Asterisks indicate superiority vs placebo (*p<0.05 **p<0.01).

The response to treatment throughout the study according to the owner assessment (CBPI) was better than for the veterinary assessment, especially in the enflicoxib treated animals, where at the end of the study 66/73 dogs responded to treatment compared to 61/77 treated with mavacoxib. In the placebo group 9/21 dogs were classified as responders. Both active treatments were always statistically superior to placebo (p<0.05), and non-inferiority was demonstrated at all times (Table 2). Fig 5 shows the treatment response rate according to the CBPI throughout the study.

**Fig 6:**
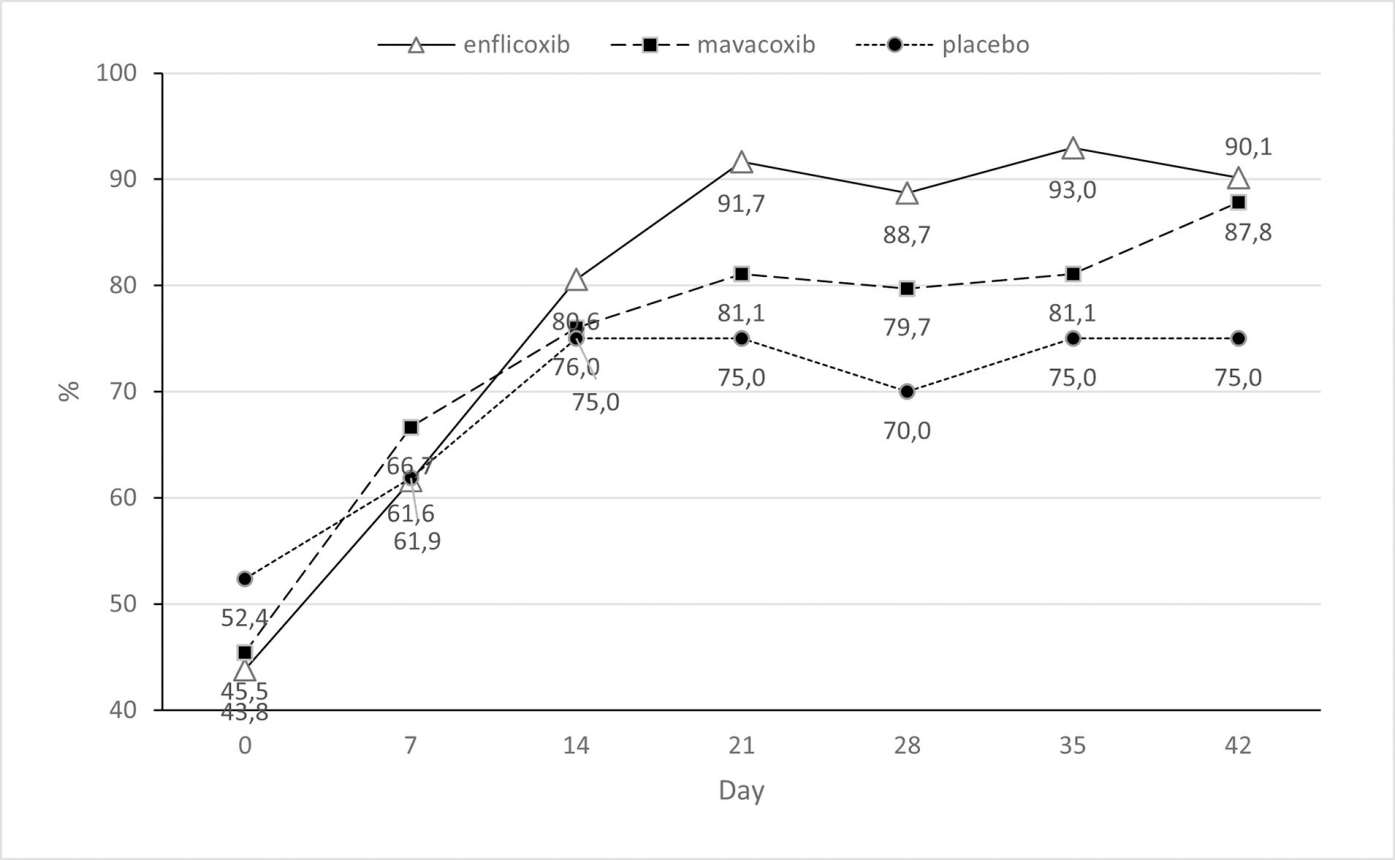
Percentage of dogs with a quality of life classified as “excellent”, “very good” or “good” in each treatment group and time point during the study. Asterisks indicate superiority vs placebo (*p<0.05 **p<0.01).

The overall owner impression of the dog’s quality of life improved in in all groups. The percentage of dogs with “good”, “very good” or “excellent” quality of life increased from 43.8 to 90.1, from 45.5 to 87 and from 52.4 to 75 in the enflicoxib, mavacoxib and placebo groups, respectively. However, statistically significant differences versus placebo were observed, only in the enflicoxib treated group, on days 21, 28 and 35 (p<0.05). Fig 6 depicts, the percentages of dogs in these three categories in each treatment group throughout the study.

When the subset of dogs with more severe clinical signs of OA at the time of inclusion (dogs with basal CSS≥8) was evaluated, all parameters showed similar results, with non-inferiority of enflicoxib compared to mavacoxib demonstrated and superiority of both active treatments versus placebo observed at similar time points.

### Safety evaluation

A total of 51 Adverse Events (AEs) in 36 dogs were reported during the study. Twenty-eight of these AEs occurred in dogs treated with enflicoxib and 23 in dogs from the mavacoxib group. According to the ABON system, a total of 13 reported AEs was categorized as “N”, and therefore excluded from further evaluation. All other AEs fell into categories”A”,”B”or”O“, as a causal relation to treatment could not be ruled out and were similarly distributed in both groups (20 in the enflicoxib and 18 in the mavacoxib groups (p = 0.783). No relationship of the observed AEs with age, sex, weight, or breed was established in any group.

Digestive tract disorders had a relatively high incidence with the main clinical signs being diarrhoea or pasty stools or vomiting (15.4% in the enflicoxib group and 13.7% in the mavacoxib group P = 0.948). All episodes (except case numbers MAS07 and RUI18) were sporadic and of mild or moderate nature and were mostly expected in a population of old dogs and not clearly related to the treatments. A description of the reported cases showing emesis, diarrhoea or soft faeces is included in Table 3.

**Table 3:** Digestive tract disorders reported as AE, classified as A, B or O.

Enflicoxib Case #	Description	Mavacoxib Case #	Description
BAZ01	Vomiting once the day after first product administration. Not treated	BUS05	Diarrhoea for one week after second product administration. Treated with antimicrobial and probiotic
BUS04	Vomited in the car on its way back home, 1–1.5 hours after first product administration. Not treated	CAR09	Emesis 15–20 minutes after second product administration. Not treated
BUS07	Bilious emesis. Three episodes after third product administration. Treated with antiemetics	DER02	Emesis once the day after first product administration, and diarrhoea for two days after second product administration. Not treated
GON03	Vomited in the car on its way back home, 1 hour after second product administration. Not treated	FER03	Diarrhoea for four days after second product administration. Not treated
MAS07	Emesis and dehydration two days before diagnosing a perforated gastric ulcer after fourth product administrations. Treated with fluids, antimicrobial, antispasmodic and antacid.	NAV07	Emesis for two days between administrations. Treated with antiulcer.
PRA08	Several episodes of emesis between second and third product administration. Associated with diarrhoea and apathy during 24hours. Not treated.	ROD04	Single vomit and loss of appetite the day of second product administration. Not treated
RUI02	Emesis with food content, once between first and second product administration. Not treated	ROD05	Soft faeces for several days at the end of the study. Not treated
ROD06	Soft faeces for two days at the end of the study. Not treated	RUI01	Diarrhoea for four days after first product administration. Not treated
SAN01	Emesis once the day of fourth product administration. Regurgitation reported on a previous day. Not treated	RUI14	Diarrhoea for two days several days after second product administration. Not treated
SAN10	Emesis once the day of fourth product administration. Loss of appetite previously reported. Not treated	RUI18	Emesis for several days after first product administration associated with haemorrhagic diarrhoea, hypothermia (36.1°c) depression and alteration of blood and renal parameters. Treated with antimicrobial, fluids, antiulcer and antiemetic
SAN17	Emesis once the day before second product administration. Not treated		

AE: Adverse Event; A = probable, B = possible, O = unclassifiable/unassessable

Other AE reported with lower incidence included more unspecific signs such as anorexia, apathy, or depression (4 cases treated with enflicoxib and 2 with mavacoxib), and single cases of constipation, leucocytosis, altered renal or blood parameters or polydipsia. All AEs recovered completely except for two Serious Adverse Events (SAEs), one in each treatment group. One case, (MAS07) treated with enflicoxib, showed emesis and dehydration together with a functional kidney disease and gastritis after the fourth treatment administration. The animal died and necropsy revealed the presence of a perforated gastric ulcer. However, the pre-existence of a subclinical process in the animal could not be ruled out. Blood levels of enflicoxib or its metabolites on the day of the AE were in the lower expected range, so the event cannot be attributed to an excessive exposure to the product. The other serious case (RUI18) received the first dose of mavacoxib and two days later suffered a haemorrhagic gastroenteritis including emesis, haemorrhagic diarrhoea and hypothermia and the animal was very depressed. Haematology revealed leucocytosis, neutrophilia, monocytosis, thrombocytopenia, high creatinine and urea levels, hypoalbuminemia, and hypercholesterolemia. The animal was euthanized but no post-mortem examination was performed (owner decision). In both cases, the signs observed are consistent with the pharmacology and the described adverse events after the use of NSAIDs.

## Discussion

The main strength of this study is that it followed the gold-standard design of being multicentred, prospective, randomized, and blind for both the veterinarian, and the owner. Given the subjective nature of some of the assessments, blinding is indeed essential for the validity of the results of this type of studies [51]. The main finding is that enflicoxib has a rapid onset of action and equivalent efficacy (statistically noninferior) and similar safety profile compared to mavacoxib, under their recommended posology. These results considered in context with the analgesic and anti-inflammatory activities of enflicoxib [35], and the inherent inflammatory component of OA, support its use for the treatment of pain and inflammation associated with OA in dogs. The relative efficacy of enflicoxib was higher than mavacoxib in most assessments and superiority over placebo was demonstrated for enflicoxib at more time points. However, no statistical differences between both treatments were seek as the study design and the sample size was not defined for this purpose. The finding that both products showed clear superiority to the placebo group in both assessments assures the presence of the disease in the study population and strengthens the efficacy of both products and the non-inferiority conclusion [52]. The inclusion of a placebo group also allows the detection of an important placebo effect, as repeatedly described for this type of studies [20, 53–56], which is observed in both the veterinary and the owner assessments.

These results confirm the findings of Salichs et al. [37], in which a tendency for a faster onset of action was also observed for enflicoxib following the same treatment schedule. The efficacy obtained in the mavacoxib group was somewhat lower than previously described by Payne-Johnson et al. [42], were mavacoxib treated dogs showed an overall improvement of 93.4% for the owner assessment and similar results for the veterinary assessment. These differences may be because although similar, the NRS used, and the definition of treatment response or improvement was different in both studies.

For the lameness and pain assessments, more objective methods such as gait analysis or force plate are available. However, these are mainly used in pilot studies with experimentally induced arthritis in a single joint. The use in this study was not possible because the study was designed as a multicentre field study to be conducted in general veterinary practices where this type of equipment is not normally available. Although, subjective NRS were used, it is acknowledged that they represent an acceptable compromise between the lack of sensitivity of simple descriptive scales and the potential limited reliability and high inter-observer variability of visual analogue scales [57]. Moreover, similar veterinary assessment tools have previously been used in the evolution of NSAIDs efficacy evaluation [39–43] and the possible subjectivity has been minimised by maintaining the veterinarian always blinded to treatment.

The clinical metrology instrument used for the owner’s assessment, the CBPI, can assess response to treatment especially in chronic pain [58, 59]. However, there is an element of inherent subjectivity which has been limited by keeping the owners also blinded to treatment to avoid any bias and by using this validated tool [44, 45]. Likewise, the treatment success criteria was previously defined by Brown et al. [55, 60] and has been recently used in the efficacy evaluation for the treatment of OA in similar studies with grapiprant [20] or bedinvetmab [21]. Interestingly, in this study, according to the owner’s assessment, enflicoxib achieved a much higher treatment success (90.4%) compared to the above-mentioned studies (48.1% and 52.6% for grapiprant and bedinvetmab, respectively).

Primary efficacy conclusions have been made based on the overall or AUC for the CSS values throughout the study. Mean group values of CSS, PSS and PIS at different time points have also been used to demonstrate efficacy. PSS results followed a pattern closer to the CSS as both scores reflect the severity of pain as assessed by the owner or the veterinarian, respectively. However, PIS results were less evident and showed less statistical significance as this score completely relies on the perception of the owner on how pain interferes with the daily life of the dog, which may be more difficult to notice. These mean group values reflect an overall response and, whilst some animals could improve notably, in other dogs this improvement could be modest or may not be even noted. In this sense, the alternative analysis performed using the individual treatment response was intended to avoid masking any lack of efficacy in some animals. This analysis of individual treatment response also showed a significant efficacy of the tested products and supported de non-inferiority conclusions. Likewise, as the severity of the disease can be an influencing factor, the efficacy of the treatments was also assessed in the subpopulation of more severely affected dogs (Basal CSS≥8). Although the number of dogs included in this population was smaller than the population calculated as minimum sample size, similar efficacy results were obtained. These results confirm the efficacy of enflicoxib treatment, at the prescribed dosage, irrespective of the stage of OA, thus confirming the results obtained by Salichs et al. [37].

Reductions in physical activity and quality of life of osteoarthritic dogs is well documented [27, 61]. In this study, better quality of life was observed with a clear improvement from baseline in all groups, with indistinguishable results during the first three weeks. These results reiterate the above-mentioned strong placebo effect also in this subjective parameter. However, despite the high improvement of the placebo group, superiority was demonstrated in the enflicoxib group in most of the assessments after the third week of treatment, while mavacoxib did not show differences at any time. The design of the study, including blinding measures also for the owner and the inclusion of a placebo group, validates and supports the efficacy of enflicoxib in improving the quality of life of the treated dogs.

Mavacoxib was selected as reference product as it has proven to be effective for the treatment of canine OA compared to other daily administered NSAIDs such as carprofen [42], or meloxicam [27]. The fact of having a long treatment interval [46] was an important factor for facilitating an effective blinding for the owners, as animals in mavacoxib group only needed 4 weekly placebo administrations to mimic the enflicoxib treatment schedule.

In this study, treatment duration may be considered short in relation to the chronicity of the disease. However, treatment duration was sufficient to show high levels of efficacy as also seen in previous studies with similar primary efficacy endpoints [42]. From the safety point of view, it has been described that longer-term therapy with NSAIDs does not increase the incidence of adverse events [23]. Indeed, the AEs observed in this study regarding incidence, type, severity, and duration were as expected and as described in other studies with daily or long acting NSAIDs [37, 42, 62–64], so no increase of AEs should be expected in longer treatments with enflicoxib, as shown in a 7-month safety laboratory study by Homedes et al. [34]. In the present study, most AEs were very mild and sporadic, and despite the prolonged half-life of the products, these AEs were completely recovered when the next dose was due, and generally, did not relapse with subsequent doses, so their causal relationship with the treatment products is not straightforward. The only severe case treated with enflicoxib was confirmed to have normal blood levels of its active metabolite so, although similar cases have been described in studies with other NSAIDs [26, 42], no definitive conclusions for the treatment as a single possible cause could be established. Moreover, in the previously published study assessing efficacy and safety of enflicoxib [37] no serious adverse events were reported when enflicoxib was administered under the same posology as described here, and the frequency of AEs and the distribution among treatments, including placebo, did not show any statistically significant difference. Therefore, the results of this study support the good safety profile of enflicoxib treatment as described in the previous overdose long term safety study with Beagle dogs [34] and in the clinical study in the target population of old dogs naturally affected with OA [37].

## Conclusions

Overall, these results confirm that enflicoxib administered weekly at 4 mg/kg with an initial loading dose of 8 mg/kg is safe and efficacious for the treatment of pain and inflammation associated to any stage of canine osteoarthritis with a faster onset of action than mavacoxib.

## Supporting information

S1 Dataset (XLSX)

## Abbreviations

AE: Adverse Event
CBPI: Canine Brief Pain Inventory
CSS: Clinical Sum Score
NRS: Numerical Rating Scale
NSAID: Non-steroidal Anti-inflammatory Drug
OA: Osteoarthritis
PIS: Pain Interference Score
PSS: Pain Severity Score
